# When Stroke Is Not a Stroke: A Case Report of Crimean-Congo Hemorrhagic Fever Presenting With Focal Neurological Deficits

**DOI:** 10.7759/cureus.115815

**Published:** 2026-09-05

**Authors:** Khuloud H Alnuaimi, Omar Farooq Al-Nahhas, Zayed Al Hammadi, Nour Abukalam

**Affiliations:** 1 Emergency Department, Sheikh Tahnoon Bin Mohammed Medical City (STMC), Al Ain, ARE

**Keywords:** camel exposure, coagulopathy, crimean-congo hemorrhagic fever, focal neurological deficits, stroke mimic, viral hemorrhagic fever

## Abstract

Crimean-Congo hemorrhagic fever (CCHF) is a potentially fatal tick-borne viral zoonosis with a wide geographic distribution, a significant case-fatality rate, and a risk of healthcare-associated transmission. Although CCHF commonly presents with nonspecific febrile illness followed by hemorrhagic manifestations, neurological symptoms may occur and can mimic acute stroke, creating diagnostic challenges in the emergency department.

We report the case of a 72-year-old male farmer in the United Arab Emirates who presented with acute slurred speech, confusion, and right-sided facial droop, prompting stroke-code activation. Initial neuroimaging showed no acute intracranial hemorrhage, established infarction, or large-vessel occlusion. The patient subsequently developed worsening tachypnea, severe metabolic acidosis, thrombocytopenia, profound coagulopathy, mucocutaneous bleeding, acute kidney injury, and multiorgan failure. CCHF was confirmed by polymerase chain reaction testing. Despite maximal supportive care, including mechanical ventilation, vasopressor support, continuous renal replacement therapy, blood product transfusion, correction of coagulopathy, ribavirin therapy, and high-level isolation precautions, the patient developed refractory hemorrhagic shock and died later the same day.

This case highlights CCHF as a rare but important stroke mimic. Emergency clinicians should consider CCHF in patients with acute neurological symptoms when accompanied by fever, bleeding manifestations, thrombocytopenia, coagulopathy, metabolic acidosis, renal dysfunction, shock, or relevant zoonotic exposure. Early epidemiological history, repeated clinical reassessment, prompt implementation of infection-control precautions, and multidisciplinary management are essential to reduce diagnostic delays and the risk of healthcare-associated transmission.

## Introduction

Crimean-Congo hemorrhagic fever (CCHF) is a potentially fatal tick-borne viral zoonosis caused by Crimean-Congo hemorrhagic fever virus (CCHFV), an enveloped, negative-sense RNA virus in the genus Orthonairovirus [1]. It has been reported in more than 30 countries across Africa, the Middle East, Central and South Asia, and southeastern Europe. CCHF remains a major public health concern because of its substantial case-fatality rate, the risk of healthcare-associated transmission, and the lack of a widely licensed vaccine or approved specific antiviral therapy [2,3].

Human infection occurs primarily through the bite of infected Hyalomma ticks or through direct contact with the blood or tissues of infected animals, especially livestock [2,4]. In the United Arab Emirates (UAE), dromedary camels are an important potential source of exposure. The detection of CCHFV-reactive antibodies and viral RNA in dromedary camels and camel-associated Hyalomma dromedarii ticks supports ongoing enzootic circulation in the region [5]. The identification of a novel CCHFV lineage and possible genomic reassortment in UAE dromedary camels further highlights the genetic diversity of the virus and underscores the need for continued One Health surveillance among individuals exposed to camels, ticks, livestock, or farms [6].

CCHF typically progresses through incubation, pre-hemorrhagic, hemorrhagic, and convalescent or fatal phases [1,2]. Early symptoms are often nonspecific and may include fever, headache, myalgia, dizziness, gastrointestinal symptoms, and constitutional complaints. Severe disease can progress to petechiae, ecchymoses, mucosal or gastrointestinal bleeding, disseminated intravascular coagulation, shock, and multiorgan failure [1,2,7]. Mortality is influenced by multiple factors, including host characteristics, viral load, route and intensity of exposure, delayed recognition, access to supportive care, and the severity of thrombocytopenia, coagulopathy, hepatic injury, and renal impairment [2,7-9].

Neurological manifestations of CCHF may include confusion, altered consciousness, seizures, dysarthria, and, less commonly, focal neurological deficits that mimic acute stroke [1,2]. Stroke-code activation is appropriate when focal deficits predominate; however, accompanying fever, bleeding manifestations, thrombocytopenia, coagulopathy, metabolic acidosis, renal dysfunction, or relevant animal exposure should prompt immediate diagnostic reassessment and infection-control precautions. Recognizing CCHF in this setting is time-critical because thrombolysis in a patient with thrombocytopenia or coagulopathy may cause catastrophic bleeding, and delayed isolation may increase the risk of healthcare-associated transmission [2,4].

We report the case of a 72-year-old camel farmer in the UAE who presented with acute dysarthria, confusion, and right-sided facial weakness, prompting stroke-code activation. His condition rapidly progressed to profound coagulopathy, multiorgan failure, refractory hemorrhagic shock, and death, with CCHF subsequently confirmed by polymerase chain reaction testing. This case highlights CCHF as a rare but important stroke mimic and underscores the value of epidemiological history, repeated clinical reassessment, prompt isolation, and multidisciplinary management when acute neurological symptoms coexist with systemic and hemorrhagic features.

## Case presentation

A 72-year-old man with hypertension and type 2 diabetes mellitus presented to the emergency department (ED) with acute slurred speech, confusion, and right-sided facial droop. The symptoms were first noted on awakening, and the last known well time was approximately 10 hours before presentation. A stroke code was activated on arrival.

Five days before presentation, he developed cough, fever, and generalized fatigue. He was evaluated at a private clinic and prescribed antibiotics. He had no recent travel history. However, he reported daily contact with dromedary camels on his farm, posing a relevant zoonotic risk for CCHF in the UAE. The precise route of zoonotic exposure was not established.

On arrival, the patient was tachypneic, with an oxygen saturation of 95% on room air. Initial vital signs are summarized in Table 1.

**Table 1:** Initial vital signs on emergency department arrival

Parameter	Result	Normal adult reference range
Blood pressure	170/80 mmHg	90/60-120/80 mmHg
Heart rate	90 beats/min	60-100 beats/min
Respiratory rate	40 breaths/min	12-20 breaths/min
Temperature	37.6°C	37.6°C
Oxygen saturation	95% on room air	95-100%

He was alert and oriented. Neurological examination demonstrated slurred speech, dysarthria, and right-sided facial weakness, without limb weakness, sensory deficit, or visual field loss. The initial National Institutes of Health Stroke Scale (NIHSS) score [10] was three, reflecting right-sided facial weakness and dysarthria. Oral petechiae, ecchymoses, and multiple bruises were also noted, raising concern for an underlying hemorrhagic process.

Non-contrast computed tomography (CT) of the brain and CT angiography were performed as part of the initial stroke evaluation. Imaging showed no evidence of acute intracranial hemorrhage, established infarction, or large-vessel occlusion, as shown in Figure 1.

**Figure 1:**
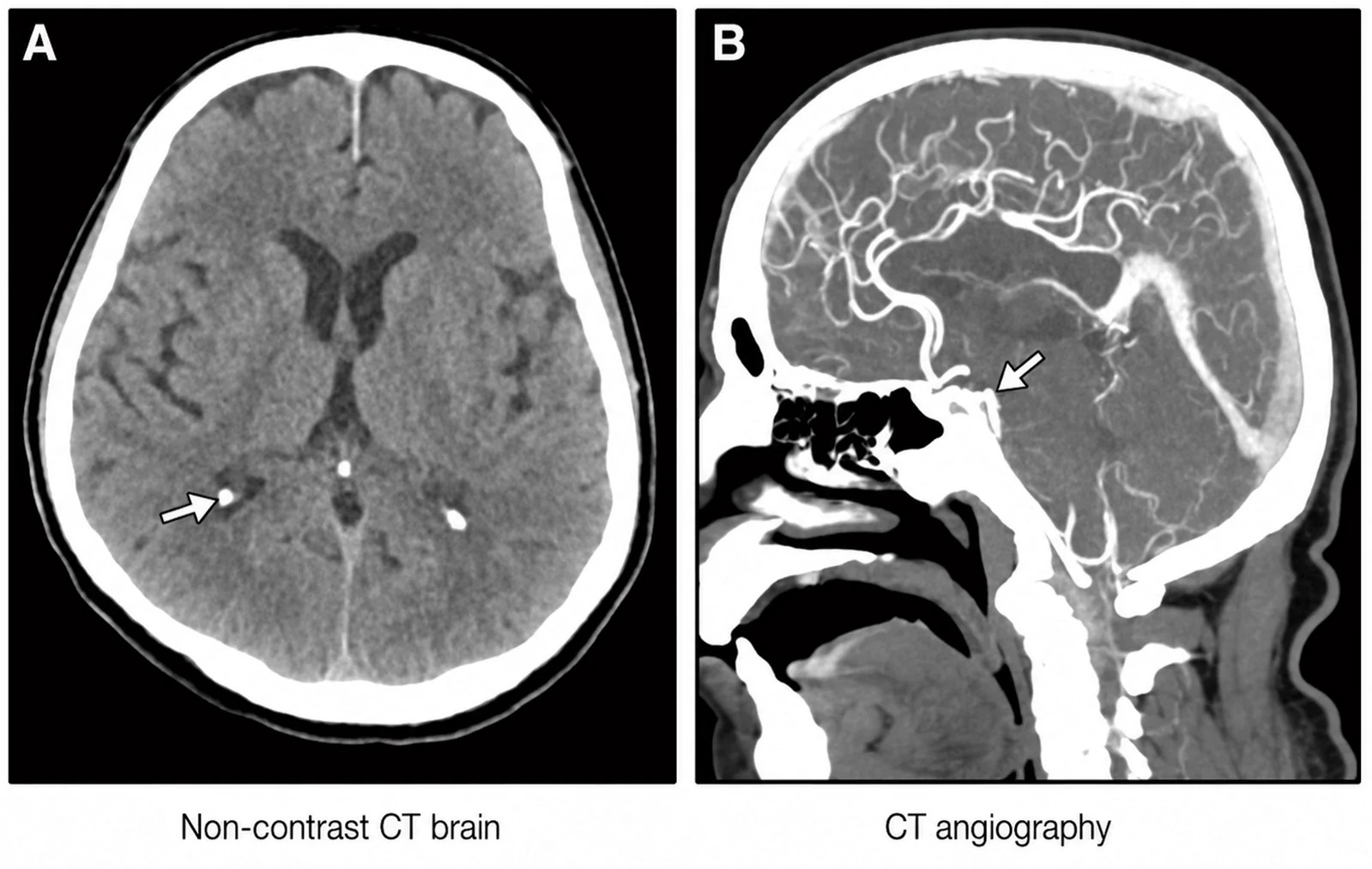
Initial neuroimaging performed during stroke evaluation (A) Non-contrast computed tomography (CT) of the brain showing no acute intracranial hemorrhage or established infarction. (B) CT angiography showing no large-vessel occlusion.

Given the approximately 10-hour interval from the last known well time, intravenous thrombolysis was not pursued. Subsequent laboratory investigations additionally demonstrated profound coagulopathy and thrombocytopenia, and the stroke code was deactivated.

At this stage, the combination of a recent febrile illness, mucocutaneous bleeding and bruising, and daily camel exposure prompted consideration of viral hemorrhagic fever, with CCHF prioritized in the differential diagnosis. During reassessment following initial neuroimaging, the patient developed worsening tachypnea. Repeat vital signs demonstrated persistent tachypnea and new tachycardia, as summarized in Table 2.

**Table 2:** Reassessment of vital signs after initial neuroimaging

Parameter	Result
Blood pressure	148/64 mmHg
Heart rate	104 beats/min
Respiratory rate	40 breaths/min
Oxygen saturation	99% on 4 L/min oxygen via a simple face mask

An initial venous blood gas obtained during early resuscitation demonstrated severe metabolic acidosis with marked hyperlactatemia and hyperkalemia, as summarized in Table 3.

**Table 3:** Initial venous blood gas analysis during early resuscitation

Parameter	Result	Reference range
pH	6.75	7.35-7.45
Partial pressure of carbon dioxide	26.5 millimeters of mercury	35-45 millimeters of mercury
Bicarbonate	Not reported	22-29 millimoles per liter
Base excess	Not reported	−2 to +2 millimoles per liter
Lactate	17.0 millimoles per liter	0.5-2.2 millimoles per liter
Potassium	6.4 millimoles per liter	3.5-5.0 millimoles per liter
Glucose	15.4 millimoles per liter	3.9-5.8 millimoles per liter
Total bilirubin	45 micromoles per liter	Less than 21 micromoles per liter
Methemoglobin	1.40%	Less than 1.5%

A repeat venous blood gas obtained approximately two hours later, following initial fluid resuscitation, bicarbonate therapy, and treatment of hyperkalemia, demonstrated persistent severe acidemia, as shown in Table 4.

**Table 4:** Repeat venous blood gas analysis after initial resuscitation

Parameter	Result	Reference range
pH	6.93	7.35-7.45
Partial pressure of carbon dioxide	26.8 millimeters of mercury	35-45 millimeters of mercury
Bicarbonate	6 millimoles per liter	22-29 millimoles per liter
Base excess	−25.7 millimoles per liter	−2 to +2 millimoles per liter
Lactate	17.0 millimoles per liter	0.5-2.2 millimoles per liter
Potassium	6.0 millimoles per liter	3.5-5.0 millimoles per liter
Sodium	142 millimoles per liter	135-145 millimoles per liter
Total bilirubin	43 micromoles per liter	Less than 21 micromoles per liter

A portable chest radiograph obtained during the initial resuscitation showed no focal airspace consolidation, pleural effusion, or pneumothorax, as shown in Figure 2.

**Figure 2:**
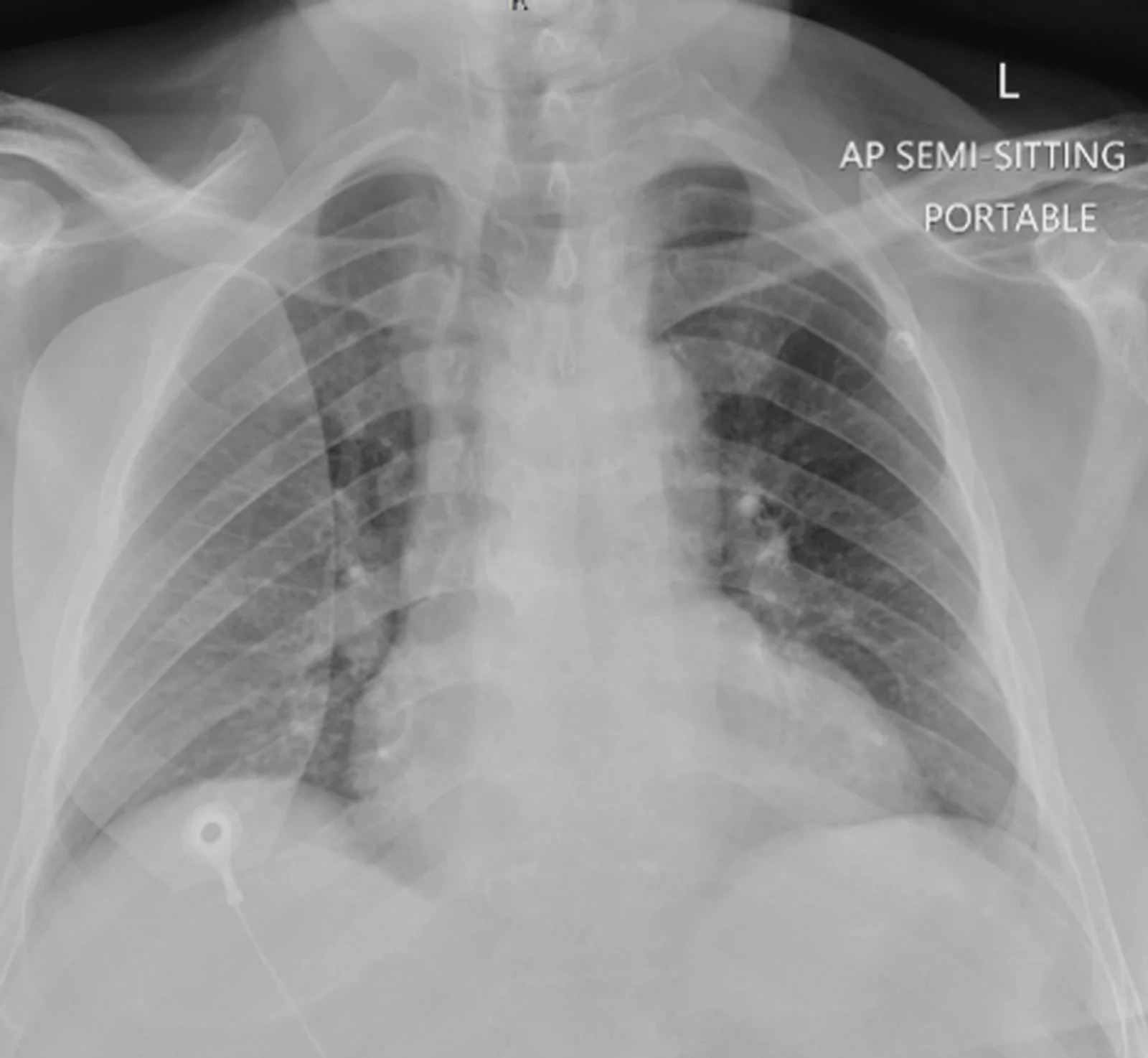
Portable chest radiograph obtained during initial resuscitation Portable anteroposterior chest radiograph showing no focal airspace consolidation, pleural effusion, or pneumothorax.

The patient was subsequently transferred to the resuscitation bay and placed under continuous cardiac monitoring. Laboratory investigations demonstrated critical coagulopathy, thrombocytopenia, severe acute kidney injury with markedly reduced estimated glomerular filtration rate, transaminitis, and markedly elevated troponin I levels, as summarized in Table 5.

**Table 5:** Emergency department laboratory investigations

Investigation	Result	Reference range
White blood cell count	16.9 × 10⁹ per liter	4.0-11.0 × 10⁹ per liter
Hemoglobin	118 grams per liter	130-170 grams per liter
Platelet count	51 × 10⁹ per liter	150-400 × 10⁹ per liter
Prothrombin time	More than 180 seconds	11-13.5 seconds
International normalized ratio	More than 15.0	0.8-1.2
Activated partial thromboplastin time	132.5 seconds	30-40 seconds
D-dimer	More than 35.2 milligrams per liter fibrinogen-equivalent units	Less than 0.5 milligrams per liter fibrinogen-equivalent units
Creatinine	749.8 micromoles per liter	60-110 micromoles per liter
Estimated glomerular filtration rate	6 mL/min/1.73 m²	>90 mL/min/1.73 m²
Alanine aminotransferase	142 units per liter	Less than 40 units per liter
Aspartate aminotransferase	589 units per liter	Less than 40 units per liter

Blood samples were notably hemolyzed, preventing formal serum electrolyte analysis.

Initial resuscitation was commenced during the first hour of ED care and included intravenous sodium bicarbonate 100 mmol, one liter of 0.9% sodium chloride, piperacillin-tazobactam 4 g intravenously, and hydrocortisone 100 mg intravenously. Hyperkalemia was treated with calcium gluconate, an insulin-dextrose infusion, and nebulized salbutamol.

Non-invasive ventilation was initiated approximately two hours after ED arrival because of worsening respiratory distress. An additional 200 mmol of intravenous sodium bicarbonate was administered during ongoing resuscitation, and prothrombin complex concentrate was given for critical coagulopathy.

The combination of rapidly progressive multiorgan failure, severe metabolic acidosis, critical coagulopathy with active bleeding from the oral cavity and rectum, thrombocytopenia, and significant camel exposure raised strong concern for viral hemorrhagic fever, with CCHF prioritized in the differential diagnosis. Infection-control precautions were escalated when viral hemorrhagic fever was suspected, particularly during high-risk procedures. The intensive care and nephrology teams were consulted, and the patient was accepted for intensive care unit admission for continuous renal replacement therapy and ongoing critical care.

Intensive care unit course

The patient was transferred to the intensive care unit approximately four hours after ED arrival. Because of worsening respiratory distress and profound hemodynamic instability, he was intubated and mechanically ventilated. Central venous and arterial access, a dialysis catheter, and an orogastric tube were inserted. He required triple vasopressor support with noradrenaline, vasopressin, and adrenaline, and continuous renal replacement therapy was initiated because of anuria and severe metabolic acidosis.

Blood product resuscitation included four units of fresh frozen plasma, two units of packed red blood cells, and one unit of apheresis platelets. Sodium bicarbonate therapy and frequent blood gas monitoring were continued. Enhanced infection-control precautions were maintained during all high-risk procedures, and the patient was managed in an isolated intensive care environment.

The infectious diseases team supported the clinical suspicion of CCHF based on the profound coagulopathy, active bleeding, multiorgan failure, and daily camel exposure. Enteral ribavirin was administered through the orogastric tube, beginning with a 2 g loading dose. The planned continuation regimen was 1 g every six hours for four days, followed by 500 mg every six hours for six days. Empirical intravenous antimicrobial therapy was also administered.

Repeat laboratory investigations demonstrated worsening multiorgan failure, severe hyperlactatemia, hypofibrinogenemia, hyperammonemia, progressive transaminitis, markedly elevated creatine kinase, and a substantial rise in troponin I, consistent with profound systemic hypoperfusion and end-organ injury (Table 6).

**Table 6:** ICU laboratory investigations during ongoing deterioration

Investigation	Result	Reference range
White blood cell count	3.7 × 10⁹ per liter	4.0-11.0 × 10⁹ per liter
Hemoglobin	81 grams per liter	130-170 grams per liter
Platelet count	48 × 10⁹ per liter	150-400 × 10⁹ per liter
Prothrombin time	22.4 seconds	11-13.5 seconds
International normalized ratio	2.13	0.8-1.2
Activated partial thromboplastin time	83.2 seconds	30-40 seconds
Fibrinogen	0.55 grams per liter	2.0-4.0 grams per liter
D-dimer	More than 35.20 milligrams per liter fibrinogen-equivalent units	Less than 0.5 milligrams per liter fibrinogen-equivalent units
Sodium	167 millimoles per liter	135-145 millimoles per liter
Creatinine	520.3 micromoles per liter	62-106 micromoles per liter
Estimated glomerular filtration rate	9 mL/min/1.73 m²	>90 mL/min/1.73 m²
Alanine aminotransferase	686 units per liter	Less than 40 units per liter
Aspartate aminotransferase	1,271 units per liter	Less than 40 units per liter
Total bilirubin	41.8 micromoles per liter	Less than 21 micromoles per liter
Troponin I	7,867.6 nanograms per milliliter	Less than 0.04 nanograms per milliliter
N-terminal pro-B-type natriuretic peptide	850.8 picograms per milliliter	Less than 300 picograms per milliliter
Procalcitonin	4.01 nanograms per milliliter	Less than 0.5 nanograms per milliliter
Lactate	27.31 millimoles per liter	0.5-2.2 millimoles per liter
Ammonia	386.2 micromoles per liter	11-32 micromoles per liter
Creatine kinase	6,092 units per liter	20-200 units per liter
Anion gap	36 millimoles per liter	8-16 millimoles per liter

Diagnosis and outcome

CCHFV reverse transcription-polymerase chain reaction (RT-PCR) testing returned positive, confirming the diagnosis of CCHF. The available laboratory documentation reported a qualitative positive result only; the specimen type, exact sampling time relative to ED arrival, and cycle threshold value or quantitative viral load were not available.

Despite maximal supportive care, including mechanical ventilation, triple vasopressor support, continuous renal replacement therapy, blood product transfusion, correction of coagulopathy, and enteral ribavirin therapy, the patient remained in refractory hemorrhagic shock. Serum lactate subsequently exceeded 30 mmol/L, reflecting profound tissue hypoperfusion.

Following multidisciplinary discussion involving the emergency medicine, intensive care, and infectious diseases teams, and after discussion with the patient’s family regarding the grave prognosis, a do-not-resuscitate order was established in accordance with institutional policy. The patient died later the same day despite maximal resuscitative efforts. The clinical course is summarized chronologically in Table 7.

**Table 7:** Timeline of clinical course from emergency department arrival ED, emergency department; NIHSS, National Institutes of Health Stroke Scale; CT, computed tomography; CCHF, Crimean-Congo hemorrhagic fever; CCHFV: Crimean-Congo hemorrhagic fever; RT-PCR: Reverse transcriptase-polymerase chain reaction.

Time from ED arrival	Key clinical events and interventions
0 hours	The patient presented with acute slurred speech, confusion, and right-sided facial droop. A stroke code was activated. Examination demonstrated dysarthria and right-sided facial weakness without limb weakness, sensory deficit, or visual field loss. The initial NIHSS score was 3. Oral petechiae, ecchymoses, and multiple bruises were noted.
0–1 hour	Non-contrast CT brain and CT angiography showed no acute intracranial hemorrhage, established infarction, or large-vessel occlusion. Given the approximately 10-hour interval from the last known well time, intravenous thrombolysis was not pursued. The stroke code was subsequently deactivated. The febrile prodrome, mucocutaneous bleeding, bruising, and daily camel exposure prompted concern for viral hemorrhagic fever, with CCHF prioritized. Initial venous blood gas analysis showed severe metabolic acidosis with hyperlactatemia and hyperkalemia. Initial resuscitation was commenced.
1–2 hours	Repeat venous blood gas analysis demonstrated persistent severe acidemia despite initial treatment. Non-invasive ventilation was initiated for worsening respiratory distress. Additional sodium bicarbonate and prothrombin complex concentrate were administered. Infection-control precautions were escalated because of concern for viral hemorrhagic fever. The intensive care and nephrology teams were consulted for organ support and continuous renal replacement therapy.
4 hours	The patient was transferred to the intensive care unit in critical condition.
6–7 hours	Endotracheal intubation was performed. Central venous access, dialysis catheter placement, arterial line insertion, and orogastric tube placement were completed. Mechanical ventilation, triple vasopressor support, and continuous renal replacement therapy were initiated. Enhanced infection-control precautions were maintained during high-risk procedures.
7–9 hours	Blood product resuscitation, sodium bicarbonate infusion, and frequent arterial blood gas monitoring continued. The infectious diseases team supported the suspected CCHF diagnosis. The patient was managed in an isolated intensive care environment with enhanced infection-control precautions. Ribavirin was commenced, and empirical broad-spectrum antimicrobials were administered.
9–10 hours	Repeat laboratory investigations demonstrated worsening multiorgan failure, severe hyperlactatemia, hypofibrinogenemia, hyperammonemia, rising troponin-I levels, transaminitis, and markedly elevated creatine kinase levels, consistent with ongoing systemic hypoperfusion and end-organ injury.
10 hours	CCHFV RT-PCR testing returned positive, confirming the diagnosis. Despite maximal supportive care, the patient remained in refractory hemorrhagic shock. Following multidisciplinary and family discussion, a do-not-resuscitate order was established in accordance with institutional policy. The patient died despite maximal resuscitative efforts.

## Discussion

CCHF usually evolves through incubation, pre-hemorrhagic, hemorrhagic, and convalescent or fatal phases [1,2]. Early symptoms are often nonspecific, but severe disease may progress to bleeding, disseminated intravascular coagulation, shock, and multiorgan failure [1,2]. In our patient, a five-day febrile illness was followed by mucocutaneous bleeding, severe coagulopathy, metabolic acidosis, renal failure, and hemodynamic instability, consistent with fulminant hemorrhagic CCHF. However, focal neurological deficits were the initial dominant feature and triggered stroke-code activation.

Neurological manifestations of CCHF include confusion, seizures, altered consciousness, and, less commonly, focal deficits that may mimic acute stroke [1,2]. Proposed mechanisms include endothelial injury, cytokine-mediated inflammation, microvascular thrombosis, intracranial hemorrhage, and toxic-metabolic encephalopathy related to shock, renal failure, hepatic dysfunction, or severe acidosis [1,7]. In this case, right-sided facial weakness, dysarthria, and confusion appropriately prompted acute stroke evaluation. The absence of intracranial hemorrhage, established infarction, or large-vessel occlusion on initial CT, together with fever, bleeding, thrombocytopenia, coagulopathy, acidosis, renal dysfunction, and camel exposure, supported an alternative systemic diagnosis while not fully excluding a small acute ischemic lesion.

When the clinical course does not fit the initial diagnosis, repeated reassessment is essential. In endemic regions, CCHF should be considered when acute neurological symptoms occur with fever, bleeding, thrombocytopenia, coagulopathy, organ dysfunction, or exposure to ticks, livestock, camels, or farms [2,4]. Daily camel exposure was an important epidemiological clue in this case, although the exact route of transmission could not be established. RT-PCR is the preferred diagnostic test during early viremia, whereas serology is more useful later because antibodies may be absent early or undetectable in fatal cases [2,4].

Infection prevention is central to suspected CCHF because transmission can occur through contact with infected blood or body fluids [2,4]. Healthcare workers are especially vulnerable during phlebotomy, airway management, and vascular access. In this case, enhanced precautions were initiated once viral hemorrhagic fever was suspected, including appropriate personal protective equipment during high-risk procedures and care in an isolated intensive care setting. Early involvement of infectious diseases, infection prevention, laboratory medicine, and public health teams is essential to reduce healthcare-associated transmission.

Management of CCHF is primarily supportive and includes hemodynamic stabilization, correction of coagulopathy, transfusion of blood products, and organ support [2,4]. Ribavirin is frequently used in severe suspected or confirmed cases, although evidence for a mortality benefit remains limited and debated [2,4,11]. Our patient received enteral ribavirin via an orogastric tube in addition to mechanical ventilation, vasopressor support, continuous renal replacement therapy, and blood product resuscitation. Because he deteriorated rapidly after admission, the effect of ribavirin could not be assessed.

The profound thrombocytopenia, active bleeding, renal failure, severe hyperlactatemia, and refractory shock reflected advanced multiorgan failure. The marked troponin I elevation likely represented shock-related myocardial injury and systemic hypoperfusion; further cardiac evaluation was not possible because of the rapid clinical decline.

This case has several limitations. Brain MRI could not be performed because of hemodynamic instability, so a small acute ischemic lesion could not be fully excluded. The exact zoonotic exposure route was also not identified. In addition, the available RT-PCR report documented a qualitative positive result only; the specimen type, sampling time, and cycle threshold value or quantitative viral load were unavailable, limiting interpretation of viral burden [9]. These limitations should be considered when interpreting this presentation as a CCHF-associated stroke mimic.

This case highlights the need to maintain rapid, protocol-driven stroke evaluation when focal deficits are present, while also reassessing for systemic red flags such as fever, bleeding, thrombocytopenia, coagulopathy, and organ dysfunction. In endemic settings, early epidemiological history, prompt isolation, CCHFV testing, and multidisciplinary collaboration are essential to guide management and reduce transmission risk.

## Conclusions

CCHF can rarely present with neurological manifestations that mimic acute stroke, leading to initial activation of stroke pathways and potential delay in recognition of an underlying viral hemorrhagic fever. Clinicians should consider CCHF in patients with acute neurological symptoms when accompanied by fever, bleeding manifestations, thrombocytopenia, unexplained coagulopathy, metabolic acidosis, renal dysfunction, shock, or relevant zoonotic exposure. Multidisciplinary collaboration among emergency medicine, infectious diseases, intensive care, neurology, nephrology, laboratory medicine, and public health is vital in managing these complex high-risk cases.
